# Transcriptome analysis of genes related to gonad differentiation and development in Muscovy ducks

**DOI:** 10.1186/s12864-020-06852-z

**Published:** 2020-06-26

**Authors:** Ding-Ping Bai, Yue Chen, Yu-Qiong Hu, Wen-Feng He, Yu-Zhu Shi, Qin-Ming Fan, Ru-Tang Luo, Ang Li

**Affiliations:** College of Animal Sciences, Fujian Agricultural and Forestry University, Fuzhou, 350002 China

**Keywords:** Muscovy ducks, Sex differentiation, Transcriptome, Sex-related genes, Gonad

## Abstract

**Background:**

Sex-related genes play a crucial role in gonadal differentiation into testes or ovaries. However, the genetic control of gonadal differentiation in Muscovy ducks remains unknown. Therefore, the objective of our study was to screen new candidate genes associated with ovarian and testicular development.

**Results:**

In this study, 24 males before gonadal differentiation (MB), 24 females before gonadal differentiation (FB), 24 males after gonadal differentiation (MA) and 24 females after gonadal differentiation (FA) were selected from Putian Muscovy ducks, forming 4 groups. RNA-Seq revealed 101.76 Gb of clean reads and 2800 differentially expressed genes (DEGs), including 46 in MB vs FB, 609 in MA vs FA, 1027 in FA vs FB, and 1118 in MA vs MB. A total of 146 signalling pathways were enriched by KEGG analysis, among which 20, 108, 108 and 116 signalling pathways were obtained in MB vs FB, MA vs MB, MA vs FA and FA vs FB, respectively. In further GO and KEGG analyses, a total of 21 candidate genes related to gonad differentiation and development in Muscovy ducks were screened. Among these, 9 genes were involved in the differentiation and development of the testes, and 12 genes were involved in the differentiation and development of the ovaries. In addition, RNA-Seq data revealed 2744 novel genes.

**Conclusions:**

RNA-Seq data revealed 21 genes related to gonadal differentiation and development in Muscovy ducks. We further identified 12 genes, namely, *WNT5B*, *HTRA3, RSPO3, BMP3, HNRNPK, NIPBL, CREB3L4, DKK3, UBE2R2, UBPL3KCMF1, ANXA2,* and *OSR1*, involved in the differentiation and development of ovaries. Moreover, 9 genes, namely, *TTN, ATP5A1, DMRT1, DMRT3, AMH, MAP3K1, PIK3R1, AGT* and *ADAMTSL1*, were related to the differentiation and development of testes. Moreover, after gonadal differentiation, *DMRT3, AMH, PIK3R1, ADAMTSL1, AGT* and *TTN* were specifically highly expressed in males. *WNT5B, ANXA2* and *OSR1* were specifically highly expressed in females. These results provide valuable information for studies on the sex control of Muscovy ducks and reveal novel candidate genes for the differentiation and development of testes and ovaries.

## Background

Sex in birds and mammals is governed by sex chromosomes [1], and sex determination genes on sex chromosomes initiate sex differentiation and regulate the differentiation of gonads into ovaries or testes [2]. A host of critical gonadal differentiation genes have been detected [3, 4]. However, screening for novel regulators of male and female embryos is highly necessary to enhance our understanding of gonadal differentiation. The duck embryo is an excellent model for studying gonadal development because development takes place in ovo, and we can intervene directly in gonadal development [5]. In ducks and other avian species, there is a female heterogametic system of ZZ/ZW sex chromosomes [6], and their sex may be governed by genes on the W chromosome [7]. Studies have found that the key Z-linked regulator of testicular morphogenesis is *doublesex and mab-3 related transcription (DMRT1)* in birds. In addition, studies have found that the mechanism of sex differentiation in birds is different from that in mammals [6, 8]. In mammals, there is a male heterogametic system of XX/XY sex chromosomes. The male is determined by a dominant gene on Y *(SRY)*, which triggers a series of events that lead to testicular development [9]. Nevertheless, the testis-determining gene, *Sex-determining Region on the Y Chromosome (SRY)*, is absent in ducks [9]. The W chromosome contains few genes and seems to have undergone independent degradation in different bird populations [10, 11]. The exact role of avian sex-determining genes on Z and W has yet to be completely resolved.

In chickens, aromatase is a key developmental switch in sex determination [12], and oestrogen synthesis by the gonad is female-specific [13]. Various enzymes are related to the biosynthesis of oestrogen [14]. At the rate-limiting step, the synthesis of oestrogens occurs via the aromatization of androgens. This reaction is catalysed by a complex of enzymes including the cytochrome P450 aromatase, the product of *the cytochrome P450 family 19 subfamily A member 1 (CYP19A1)* gene, and a NADPH-dependent cytochrome P450 reductase in a wide variety of tissues, including ovary and testis tissue [15–17]. In addition, aromatase inhibitor can induce female conversion into males in Muscovy ducks [18]. Thus, aromatase may play a role in testicular and ovarian differentiation in Muscovy ducks.

In Muscovy duck embryos, the nephridium appears in a transverse section at E4. In that area, 3–6 layers of cells are observed from the splanchnic layers to the coelom corner. The genital ridge is observed at E6. At E7 and E8, the initial shape of the gonad emerges. At E9, gonads initiate differentiation into spermatogonia or spermatocytes. In addition, several identified genes show sexually dimorphic expression, such as *DMRT1, anti-Mullerian hormone (AMH),* and *oestrogen receptor-α (ESR-α)* [19]. However, in Muscovy ducks, the sex differentiation mechanisms underlying reproductive regulation remain unknown.

RNA-Seq provides general knowledge about the complete set of transcripts in a particular cell and identifies a number of phenotype-associated DEGs. Research in chickens has shown that the robust sexually dimorphic gene expression of both sex-linked and autosomal genes was detected by RNA-Seq in tissues pre-dating gonadogenesis [20]. In domestic ducks, several candidate genes involved in the development of plumulaceous feather and flight feather structures were obtained by RNA-Seq [21]. Research in Peking ducks has revealed that a key regulator of duck follicle development in the laying period [3]. In addition, RNA-Seq is also widely used to explore the mechanism of sex differentiation. For example, research in chickens revealed that the left-right asymmetry mechanism could be detected in female gonads by RNA-Seq [22]. Researchers have focused on sex differentiation in chickens, but transcriptomic analysis of the sex differentiation mechanism in Muscovy ducks has rarely been reported. Therefore, we used RNA-Seq to study the sex differentiation mechanism of Muscovy ducks and analysed the mRNA expression levels of gonadal tissues at two developmental time points in female and male Muscovy ducks. This study allowed us to further unravel the transcriptome of Muscovy ducks and provided valuable information for studying sex control in Muscovy duck.

## Results

### Overview of RNA-Seq results

**Fig. 1:**
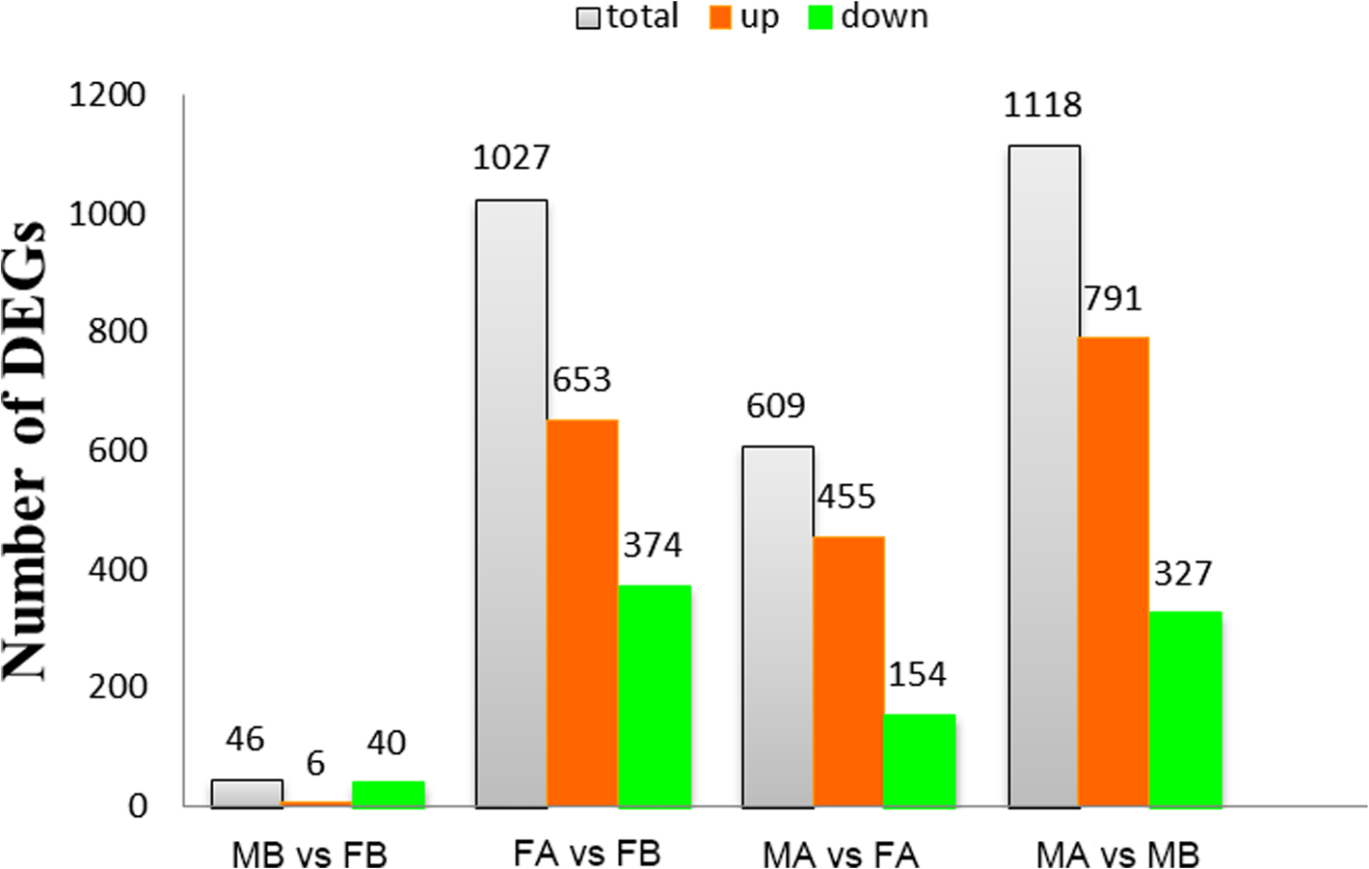
The number of differentially expressed genes between the comparison libraries. Total DEGs (grey), up-regulated genes (red), and down-regulated genes (green) are presented by a histogram

In order to identify gonadal differentiation genes in Muscovy ducks, transcriptome analysis of Muscovy duck gonads was performed on MB, FB, MA, and FA. The main RNA-Seq results are listed in Tables 1, Tables 2, and Fig. 1. RNA-Seq yielded from 47,282,572 to 70,602,322 clean reads with more than 89% Q30 bases from each library; 47.36–52.117% of the clean reads were uniquely mapped to the *Anas platyrhynchos* genome (URL:https://www.ncbi.nlm.nih.gov/genome/?term=Anas+platyrhynchos). The mapping rate was much lower than that of chickens (79%) [23]. It was similar to Peking duck (42.27%) [24] and Jinding duck (55.9%) [25]. Given that RNA-Seq produced 47,282,572 to 70,602,322 clean reads, as shown by the percentage of Q30 bases (Table 1), we suspected that the main reason for the low mapping rates might be the poor quality of the currently published duck reference genome assembly [26]. However, on the basis of the criteria reported by Conesa [27] and Martin [28], the number of mapped reads was still sufficient to reconstruct intact transcripts and reliably quantify expression levels for most medium-abundance and high-abundance genes [25]. The gene structure of each library was analysed, and approximately 87% of the mapped reads were aligned to the exon region of the *Anas platyrhynchos* genome, followed by the intergenic and intron regions (Table 2).

**Table 1:** Summary of the RNA-Seq data collected from MB, FB, MA and FA

Sample name	Raw reads	Clean reads	Clean bases	Error rate (%)	Q20 (%)	Q30 (%)	GC content (%)
FB1	73,297,772	70,602,322	10.59G	0.02	95.93	90.23	49.71
FB2	60,975,078	58,772,554	8.82G	0.02	95.84	89.93	50.49
FB3	59,397,884	57,157,288	8.57G	0.02	95.50	89.34	49.85
MB1	52,554,762	51,693,516	7.75G	0.02	96.64	91.85	51.42
MB2	62,138,482	59,945,236	8.99G	0.02	95.04	88.28	50.22
MB3	55,490,528	54,592,060	8.19G	0.02	96.71	91.92	50.50
FA1	58,175,840	56,009,556	8.40G	0.02	95.68	89.64	51.50
FA2	50,554,446	48,793,882	7.32G	0.02	95.61	89.44	51.29
FA3	61,510,464	59,329,652	8.90G	0.02	95.52	89.26	50.95
MA1	48,763,588	47,282,572	7.09G	0.02	95.57	89.32	51.52
MA2	58,272,482	57,132,880	8.57G	0.02	97.03	92.59	51.42
MA3	58,878,702	57,142,740	8.57G	0.02	95.52	89.25	50.96

**Table 2:** Summary of clean reads mapped from MB, FB, MA and FA to the reference genome

Sample name	Total reads	Total mapped	Multiple mapped	Uniquely mapped	Reads mapped to ‘+’	Reads mapped to ‘-’
FB1	70,602,322	34,506,109 (48.87%)	593,713 (0.84%)	33,912,396 (48.03%)	17,038,856 (24.13%)	16,873,540 (23.9%)
FB2	58,772,554	29,828,795 (50.75%)	519,611 (0.88%)	29,309,184 (49.87%)	14,715,210 (25.04%)	14,593,974 (24.83%)
FB3	57,157,288	27,739,072 (48.53%)	471,088 (0.82%)	27,267,984 (47.71%)	13,689,588 (23.95%)	13,578,396 (23.76%)
MB1	51,693,516	25,653,470 (49.63%)	426,958 (0.83%)	25,226,512 (48.8%)	12,670,333 (24.51%)	12,556,179 (24.29%)
MB2	59,945,236	30,339,732 (50.61%)	510,473 (0.85%)	29,829,259 (49.76%)	14,955,979 (24.95%)	14,873,280 (24.81%)
MB3	54,592,060	28,392,840 (52.01%)	473,745 (0.87%)	27,919,095 (51.14%)	14,024,427 (25.69%)	13,894,668 (25.45%)
FA1	56,009,556	27,021,161 (48.24%)	493,654 (0.88%)	26,527,507 (47.36%)	13,317,028 (23.78%)	13,210,479 (23.59%)
FA2	48,793,882	24,294,041 (49.79%)	453,668 (0.93%)	23,840,373 (48.86%)	11,963,536 (24.52%)	11,876,837 (24.34%)
FA3	59,329,652	28,914,663 (48.74%)	525,602 (0.89%)	28,389,061 (47.85%)	14,257,697 (24.03%)	14,131,364 (23.82%)
MA1	47,282,572	24,015,491 (50.79%)	433,496 (0.92%)	23,581,995 (49.87%)	11,827,474 (25.01%)	11,754,521 (24.86%)
MA2	57,132,880	30,326,910 (53.08%)	553,848 (0.97%)	29,773,062 (52.11%)	14,950,119 (26.17%)	14,822,943 (25.94%)
MA3	57,142,740	28,317,706 (49.56%)	498,305 (0.87%)	27,819,401 (48.68%)	13,965,413 (24.44%)	13,853,988 (24.24%)

### Significantly differentially expressed transcripts and clustering

**Fig. 2:**
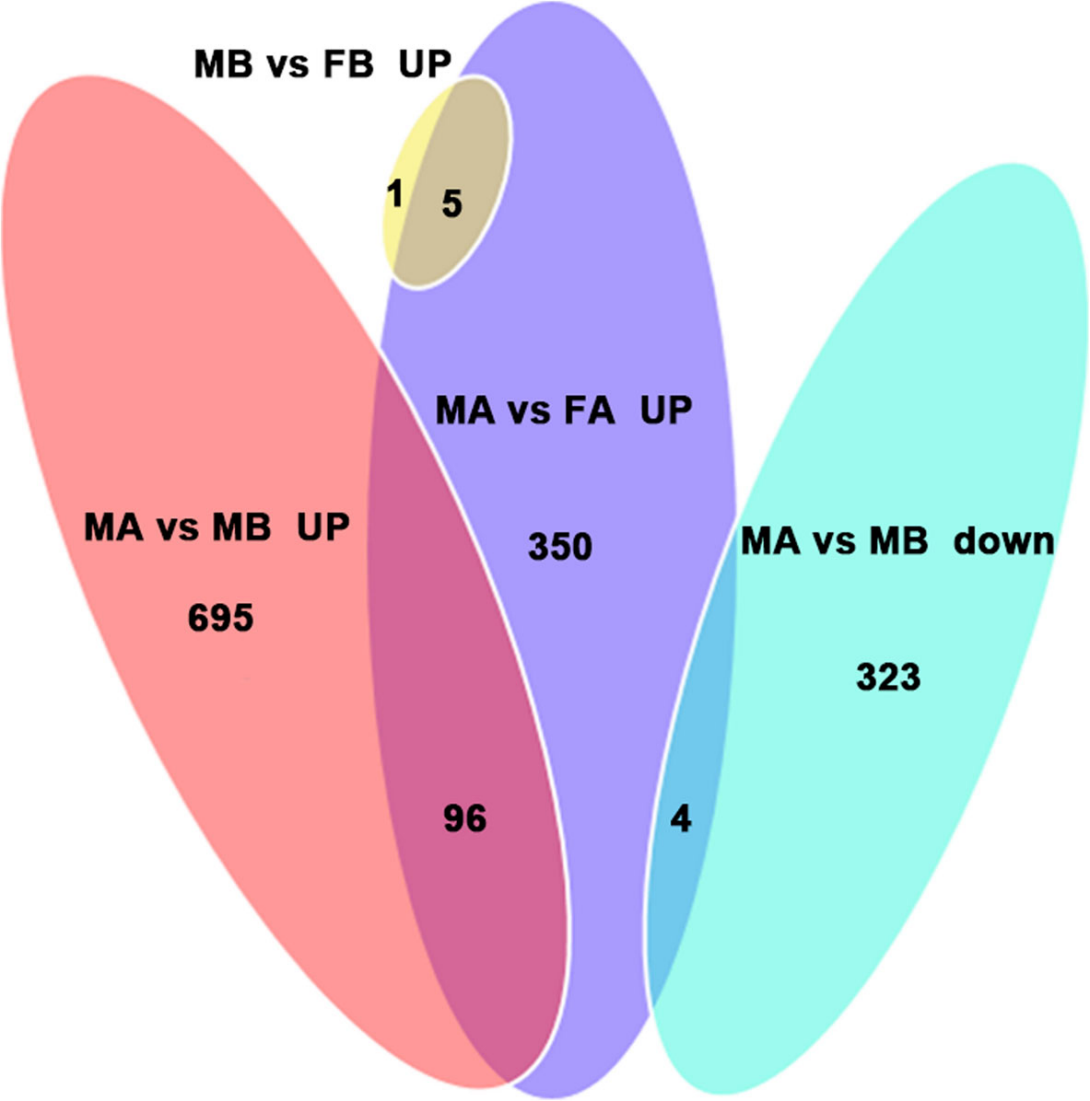
Venn diagram of male-related differentially expressed genes

**Fig. 3:**
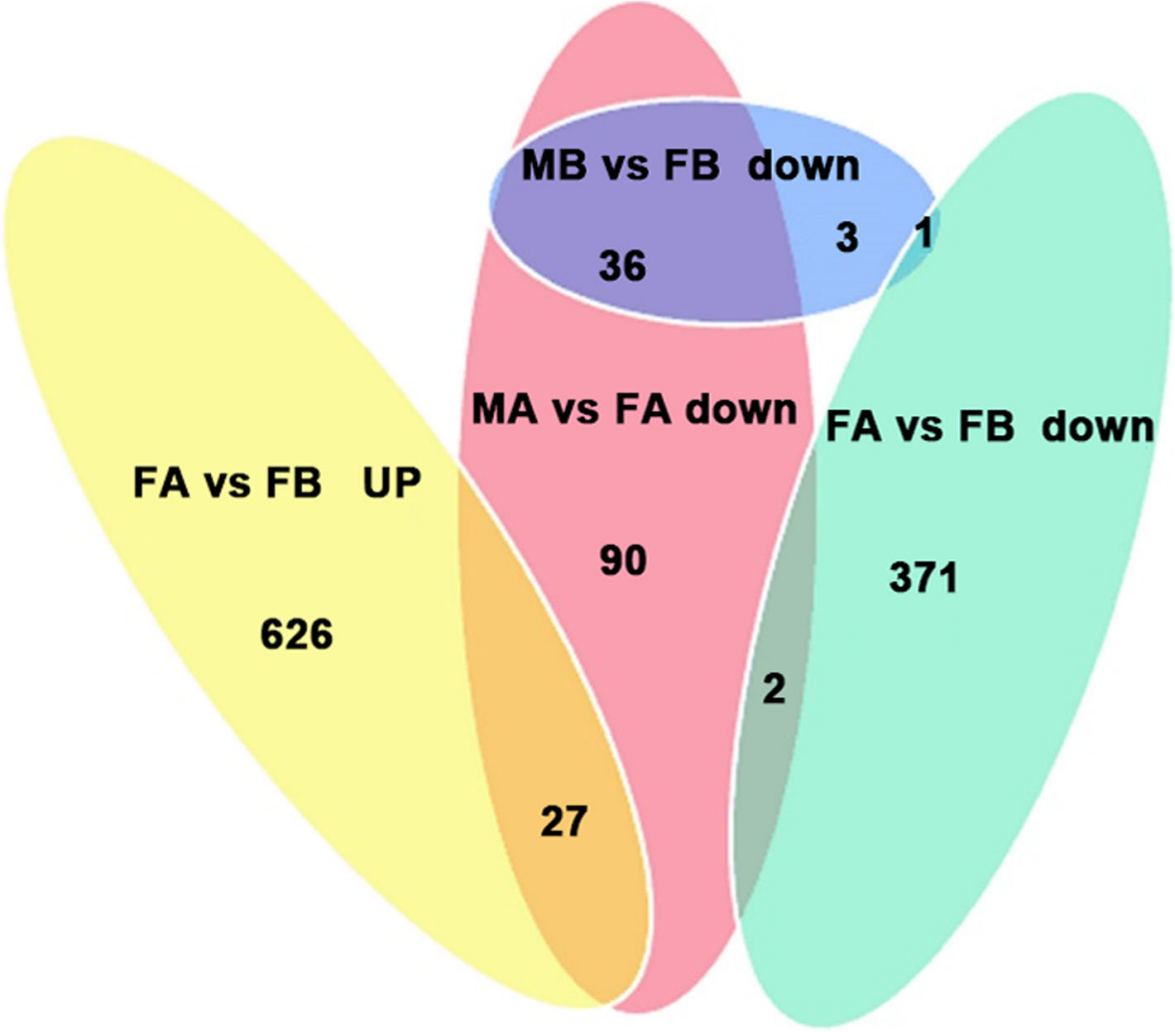
Venn diagram of female-related differentially expressed genes

**Fig. 4:**
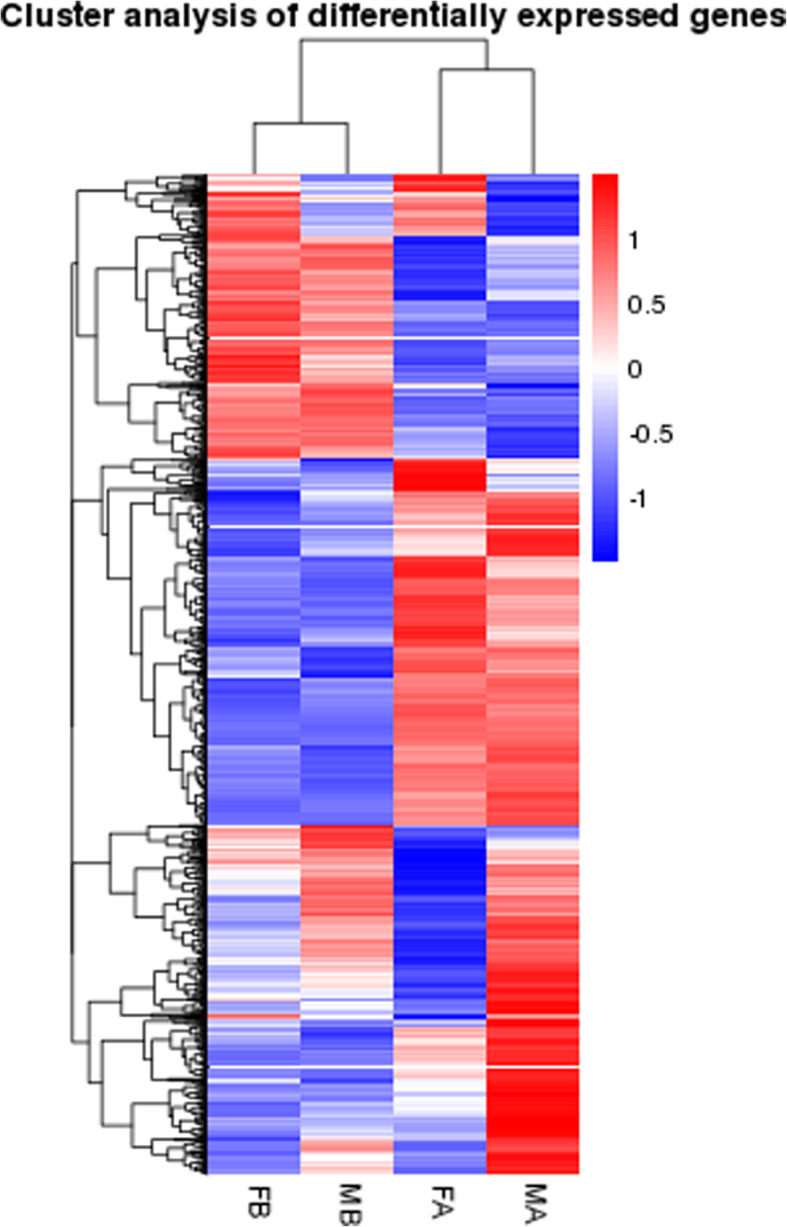
Heat map analysis is used to classify gene expression patterns at MB, FB, MA, and FA. Genes with similar expression patterns were clustered, as shown in the heat map. Red represents genes with high expression levels and blue represents genes with low expression levels

In the comparison of MB vs FB, 40 DEGs were down-regulated and 6 DEGs were up-regulated. In the comparison of FA vs FB, 374 DEGs were down-regulated, and 653 DEGs were up-regulated. In the comparison of MA vs FA, 154 DEGs were down-regulated, and 455 DEGs were up-regulated. In the comparison of MA vs MB, 327 DEGs were down-regulated, and 791 DEGs were up-regulated. This result may be due to the dimorphic expression of sex differentiation genes after gonadal differentiation (Fig. 1). To more precisely examine DEGs, a Venn diagram analysis revealed a total of 350 upregulated genes in MA vs FA, such as *ATP synthase, H+ transporting, mitochondrial F1 complex, alpha subunit 1 (ATP5A1)*, *doublesex and mab-3 related transcription factor 3 (DMRT3), AMH, a disintegrin and metalloproteinase with thrombospondin motif like 1 (ADAMTSL1), titin (TTN), phosphoinositide-3-kinase regulatory subunit 1 (PIK3R1), angiotensinogen (AGT), DMRT1 and mitogen-activated protein kinase kinase kinase 1 (MAP3K1)*. Among these, *ATP5A1* was upregulated in both the MB vs FB and MA vs FA comparisons. A total of 96 genes were upregulated in both the MA vs FA and MA vs MB comparisons, including *DMRT3, AMH, ADAMTSL1, PIK3R1 and TTN* (Fig. 2). By comparing the downregulated genes in MB vs FB with those in MA vs FA, 36 identical genes were identified, including *heterogeneous nuclear ribonucleoprotein K (HNRNPK), nipped-B-like protein (NIPBL), cyclic AMP-responsive element-binding protein 3-like protein 4 (CREB3L4), ubiquitin-conjugating enzyme E2 R2(UBE2R2) and E3 ubiquitin-protein ligase KCMF1 (UBL3KCMF1)*. In addition, the comparison between the downregulated genes in MA vs FA and the upregulated genes in FA vs FB revealed a total of 27 identical genes, among which *HtrA serine peptidase 3 (HTRA3), odd-skipped related transcription factor 1 (OSR1), Wnt family member 5B (WNT5B), and annexin A2 (ANXA2)* were identified (Fig. 3). Hierarchical clustering was performed in MB, FB, MA and FA. The expression level of DEGs did not greatly change between FB and MB. This finding indicates that many sex-related genes have not yet been expressed prior to sex differentiation. Interestingly, the DEGs produced different clustering results in MA vs FA, which showed sexually dimorphic expression (Fig. 4).

### GO and KEGG enrichment analysis of DEGs

**Fig. 5:**
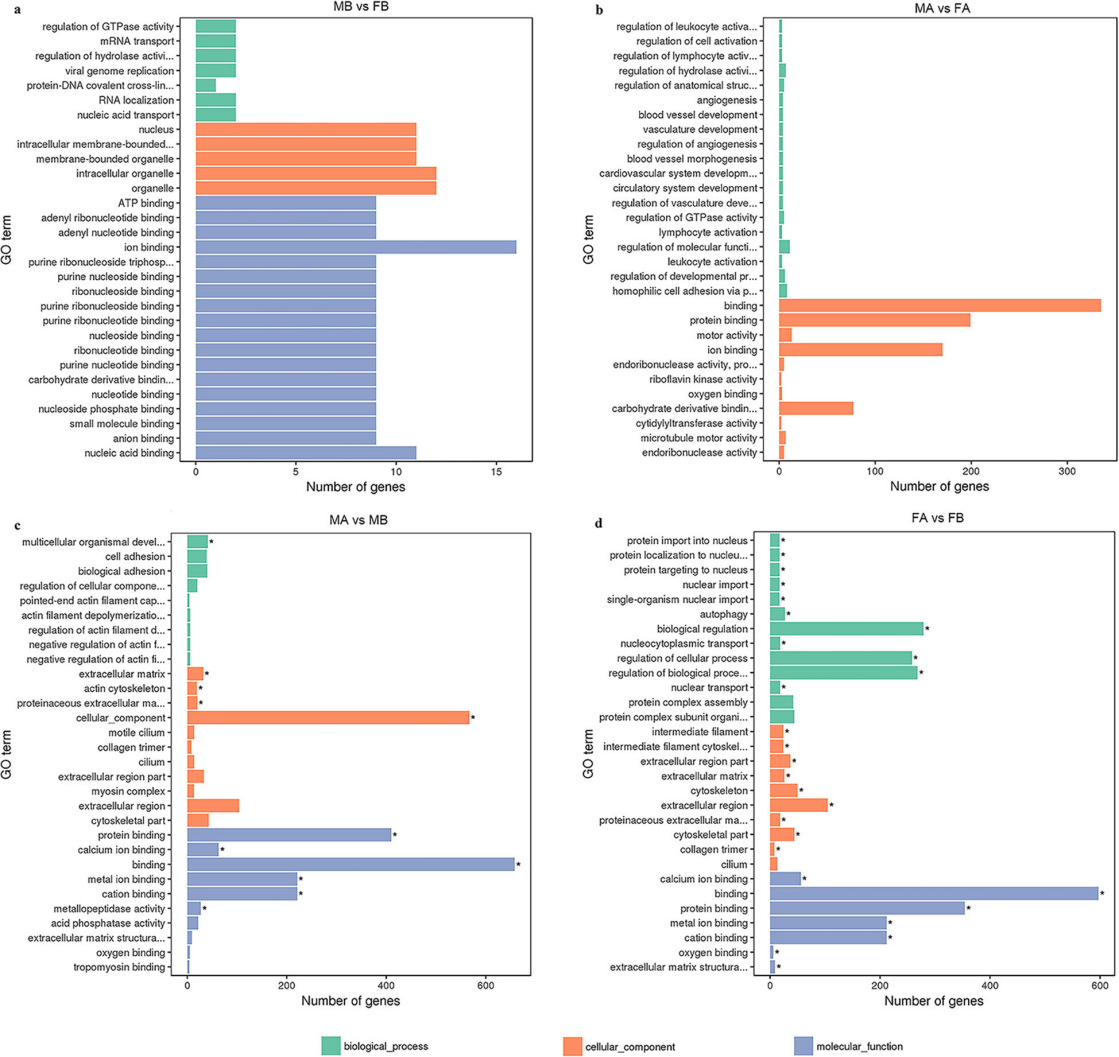
GO enrichment terms of differentially expressed genes. **a** MB-vs-FB; **b**. MA-vs-FA; **c**. MA-vs-MB; **d**. FA-vs-FB. The results are summarized in three main categories: biological process (BP), cellular component (CC) and molecular function (MF). The x-axis indicates the number of genes, and the y-axis indicates the second-level GO term

To better understand the possible functions involved in gonad development in Muscovy ducks, the DEGs from MB, FB, MA, and FA were annotated with the GO database, and the genes fell into three categories: biological process (BP), cellular component (CC) and molecular function (MF). According to the GO enriched results, *dickkopf WNT signalling pathway inhibitor 3 (DKK3)* was enriched in the regulation of the female receptivity process, *bone morphogenetic protein 3 (BMP3)* was enriched in germ cell development, oogenesis, and the female gamete generation process. *TTN* was enriched in the male gamete generation, spermatogenesis, sperm individualization process, and germ cell development process. *AMH* was enriched in the sex differentiation process. *AGT* was enriched in the fertilization process. *OSR1* and *ATP5A1* were enriched in the cellular aromatic compound metabolic process. *NIPBL*, *HNRNPK, ANXA2, UBL3KCMF1* and *CREB3L4* were enriched in the aromatic compound biosynthetic process. To identify genes useful for further study, 30 significant GO terms are listed in Fig. 5.

**Fig. 6:**
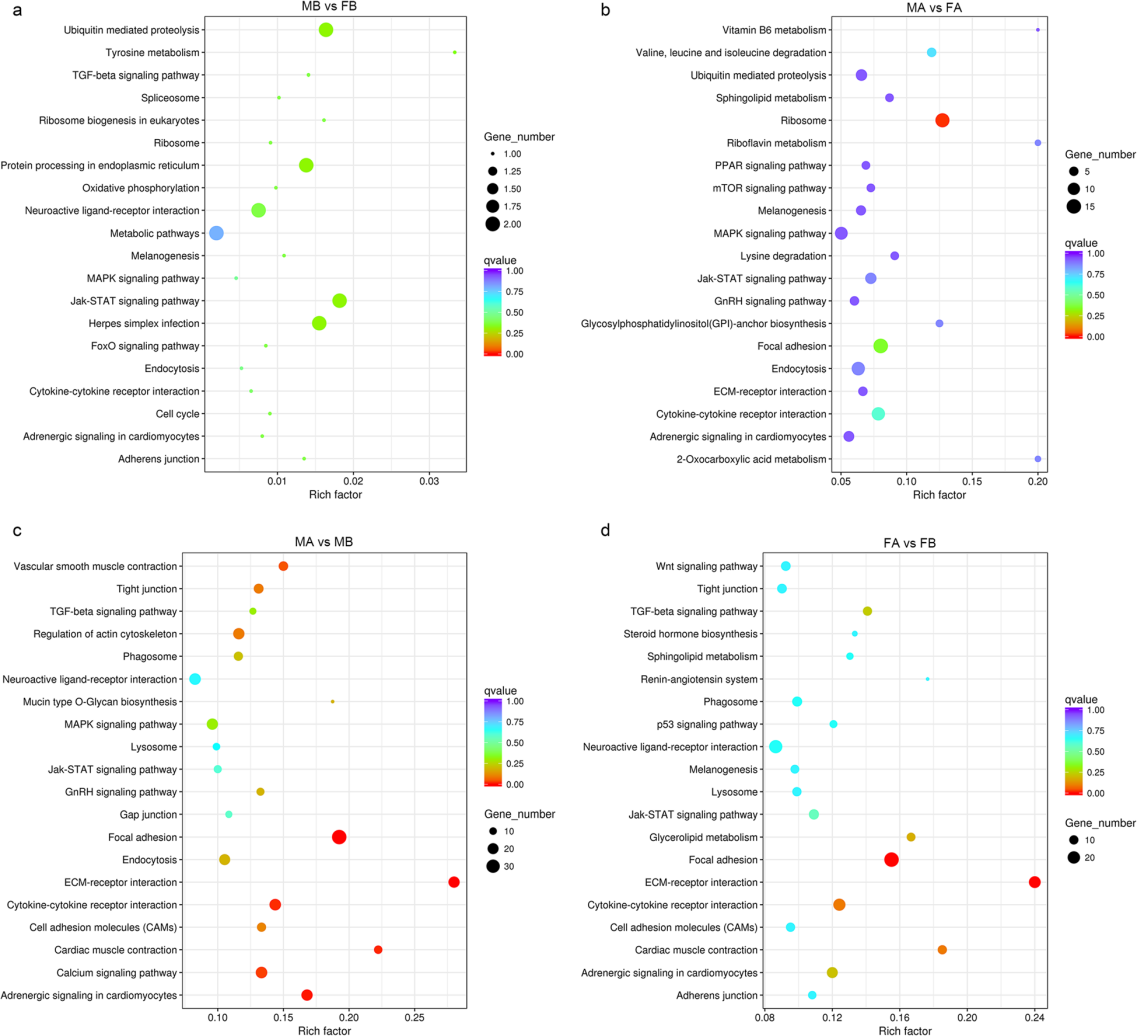
Top 20 pathways in KEGG enrichment by QValue. **a**. MB-vs-FB; **b**. MA-vs-FA; **c**. MA-vs-MB; **d**. FA-vs-FB. The Rich factor is the ratio of the differentially expressed number of genes in the pathway and the total number of genes in the pathway. The higher the Rich factor, the higher is the degree of enrichment. The QValue is the *P*-value after multiple hypothesis test correction, in the range from 0 to 1; the closer the QValue is to zero, the more significant is the enrichment

Subsequently, KEGG pathway analysis was performed, revealing 20, 108, 108 and 116 signalling pathways enriched in MB vs FB, MA vs MB, MA vs FA, and FA vs FB, respectively. The most enriched top 20 KEGG pathways are listed in Fig. 6. In MB vs FB, the most enriched KEGG pathways were ubiquitin mediated proteolysis, herpes simplex infection, the Jak-STAT signalling pathway, protein processing in the endoplasmic reticulum, neuroactive ligand-receptor interaction, and metabolic pathways (Fig. 6a). In MA vs FA, the six most enriched pathways were metabolic pathways, cytokine-cytokine receptor interaction, focal adhesion, ribosome, endocytosis, and MAPK signalling pathway (Fig. 6b). Most of the top enriched KEGG pathways were focal adhesion, adrenergic signalling in cardiomyocytes, cytokine-cytokine receptor interaction ECM-receptor interaction, and cardiac muscle contraction in MA vs MB (Fig. 6c). In FA vs FB, the six most enriched pathways were ECM-receptor interaction, metabolic pathways, cytokine-cytokine receptor interaction, adrenergic signalling in cardiomyocytes, focal adhesion, and neuroactive ligand-receptor interaction (Fig. 6d). *BMP3* was enriched in the cytokine-cytokine receptor interaction signalling pathway. *AMH* was enriched in the TGF-β signalling pathway and the cytokine-cytokine receptor interaction. *AGT* was enriched in adrenergic signalling in cardiomyocytes, the neuroactive ligand-receptor interaction and the vascular smooth muscle contraction signalling pathway. *ATP5A1* was enriched in the oxidative phosphorylation and metabolic pathways, and *MAP3K1* was enriched in the MAPK signalling pathway.

### Screening of gonadal differentiation genes

**Fig. 7:**
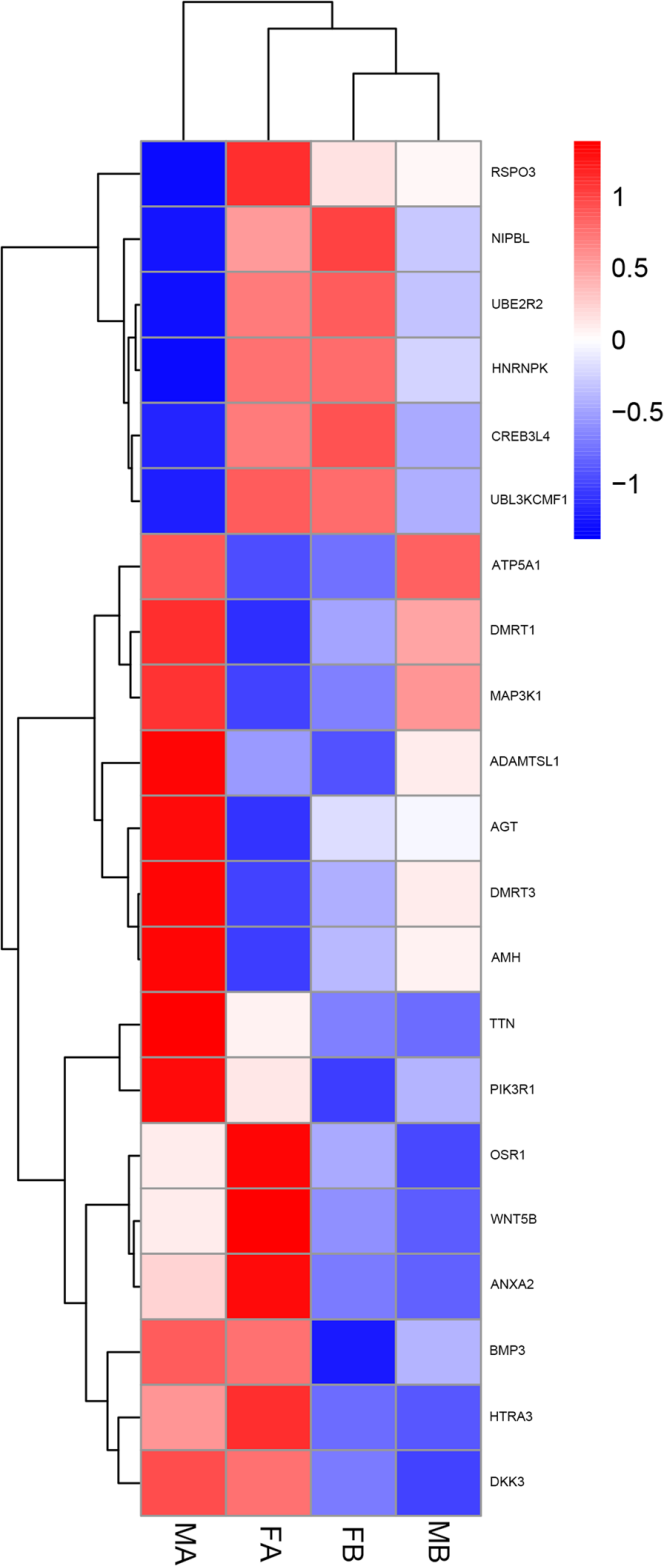
Heat map diagram of expression levels for selected sex-related genes. Red represents a high expression level, and blue indicates a low expression level. The columns and rows in the heat map represent samples and genes, respectively. Sample names are displayed below the heat map. The colour scale indicates fold changes in gene expression

**Fig. 8:**
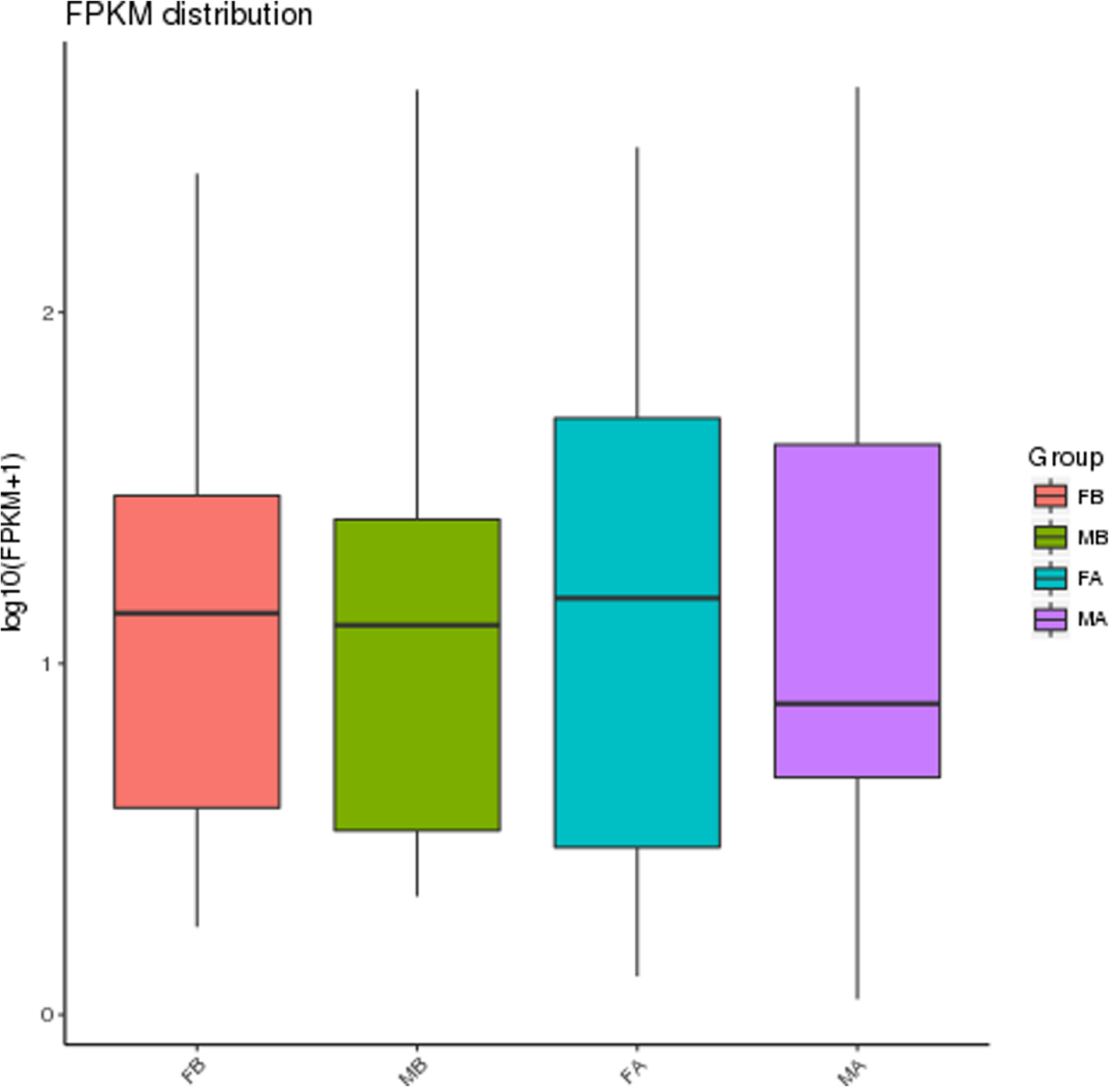
The x-axis represents the group name, whereas the y-axis represents log10 (FPKM+ 1) values. Box plots indicate the maximum, top quartile, median, bottom quartile, and minimum values from top to bottom

Of the DEGs, 21 DEGs were identified as sex-related genes according to clustering, GO analyses, and the published literature. The majority were unknown in Muscovy ducks, but most had already been identified in other organisms. According to the clustering diagram (Fig. 4), considerable changes in gene expression occurred in MA vs FA. We further excavated DEGs from the MA and FA groups, and we revealed that *DMRT1*, *DMRT3, AMH, MAP3K1, ADAMTSL1, ATP5A1, AGT, PIK3R1* and *TTN* were significantly upregulated in MA vs FA. In addition, *NIPBL, HNRNPK, UBE2R2, UBL3KCMF1, CREB3L4, R-spondin 3(RSPO3), WNT5B, HTRA3, ANXA2* and *OSR1* were significantly downregulated in MA vs FA. These genes were categorized into 10 groups putatively involved in oogenesis, gametogenesis, sex differentiation, aromatic compound biosynthetic process, female receptivity, spermatogenesis, sperm individualization process, cellular aromatic compound metabolic process, fertilization, and germ cell development process. In addition, *DKK3* and *BMP3* were significantly upregulated in FA vs FB, and *DKK3* was enriched in the regulation of female receptivity process. *BMP3* was enriched in the oogenesis and female gamete generation process. Among these DEGs, *DMRT1, DMRT3, AMH, PIK3R1, MAP3K1, ADAMTSL1, ATP5A1, AGT* and *TTN* were involved in the differentiation and development of the testes, and *NIPBL, HNRNPK, UBE2R2, UBL3KCMF1, CREB3L4, RSPO3, WNT5B, HTRA3, ANXA2, DKK3, BMP3* and *OSR1* were involved in the differentiation and development of the ovaries. Hierarchical clustering was performed to compare these genes in FA, FB, MA, and MB (Fig. 7), and genes with similar expression patterns were classified into the same group. *DMRT3, AMH, PIK3R1, ADAMTSL1, AGT* and *TTN* were classified into MA, and *WNT5B, ANXA2* and *OSR1* were classified into FA. Therefore, after gonadal differentiation, *DMRT3, AMH, PIK3R1, ADAMTSL1, AGT* and *TTN* were specifically highly expressed in males. *WNT5B, ANXA2* and *OSR1* were specifically highly expressed in females. The distribution of gene expression levels between different groups is shown by boxplots (Fig. 8).

### Verification of DEGs by qPCR

**Fig. 9:**
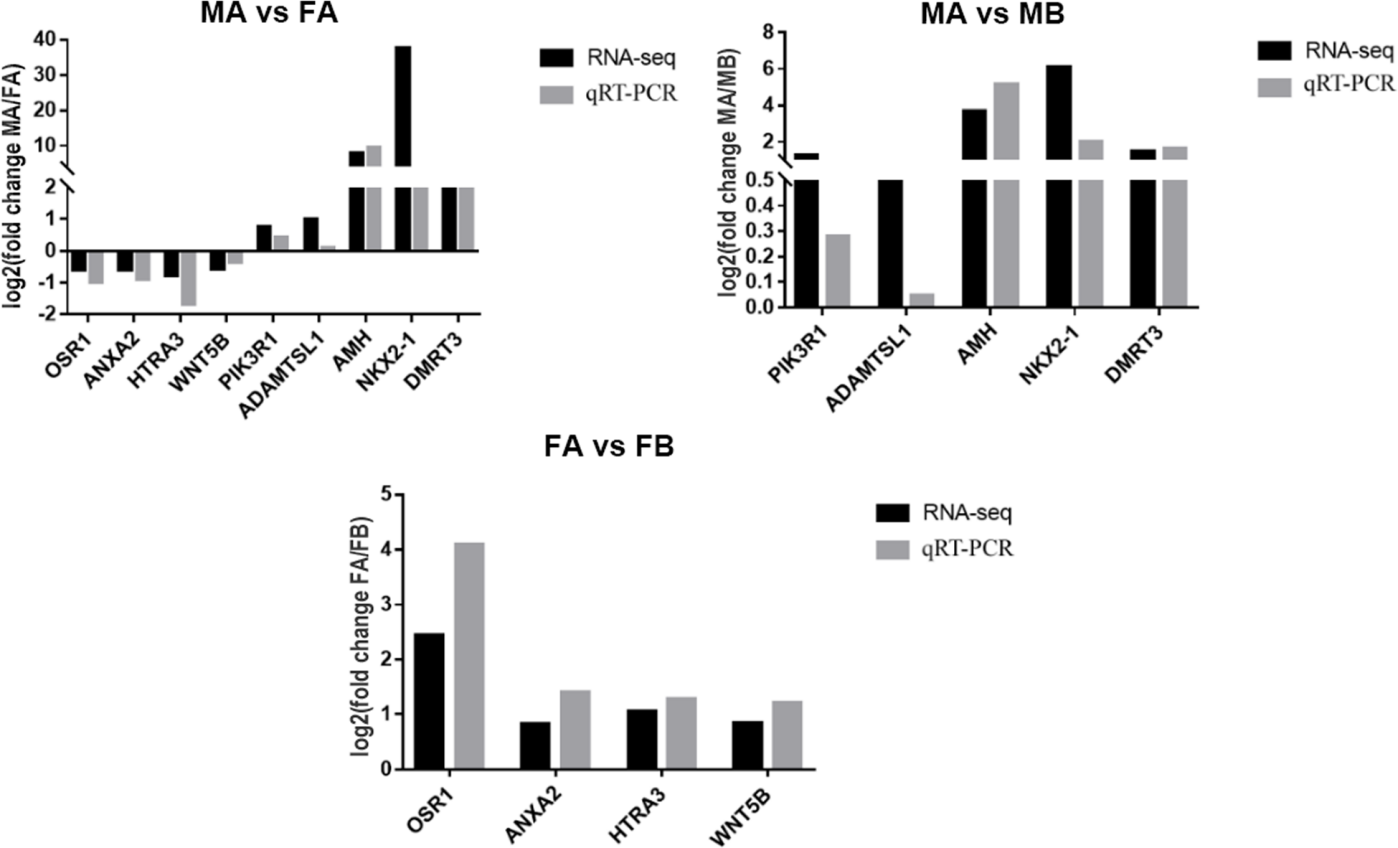
qRT-PCR validation of differentially expressed genes identified by RNA-Seq

Nine genes were selected for RT-qPCR validation to verify the reproducibility of RNA-Seq results. Among these, 5 genes were upregulated in both the MA vs FA and MA vs MB comparisons, and 4 genes were downregulated in the MA vs FA comparison but upregulated in the FA vs FB comparison. The expression profiles of the nine candidate genes generated from RT-qPCR corresponded to the RNA-Seq results (Fig. 9). The primers used for the 9 genes are listed in Table 3.

**Table 3:** PCR primers used in this study

Genes	Sequence (5′-3′)	Product size (bp)	GenBank No.
OSR1	ACCGATGAACGACCCTACAC CCTTTCCCACATTCTTGACAC	125	XM_027455274.1
ANXA2	AATCATCTGCTCACGGACAAAT GTCGATCACAGAAGTATCTTCACAT	172	XM_027466033.1
HTRA3	CCTCTAGTTAATCTGGATGGTGAAG TGATGCGGTCTGATGGAATAG	91	XM_021270005.2
PIK3R1	GGAACGTGGGAAATATCAACAG ACTTCTCCATCCACCACAACA	130	XM_027446161.1
ADAMTSL1	GCAGGAAAGCCGCTAATAAAG GCAGGTGGTGAGCAGAGGAT	199	XM_027446720.1
AMH	AGTGCGGCTGTCGGTAGAG GGGAAAAGACGTGCGGAG	99	AY904047.1
NKX2–1	ACCCCGACTCAGGTCAAAAT CATTGGTAGCCACTGTGATTGT	253	XM_027458955.1
DMRT3	CTGTCCTTCCTTCTCGCTCAT GCTGCACTGCAAATATCACTTAG	197	XM_027446949.1
WNT5B	CTGCTTTCACCTATGCTGTCA ACCCGTACTCCACGTTATCC	165	XM_027467202.1
β-actin	GCTATGTCGCCCTGGATTT CCACAGGACTCCATACCCAA	169	EF667345.1

### Prediction of novel transcripts

From the sequencing data, 2744 novel genes were detected by genome assembly and compared with the reference genome. A list of results is shown in Additional file 1.

## Discussion

**Fig. 10:**
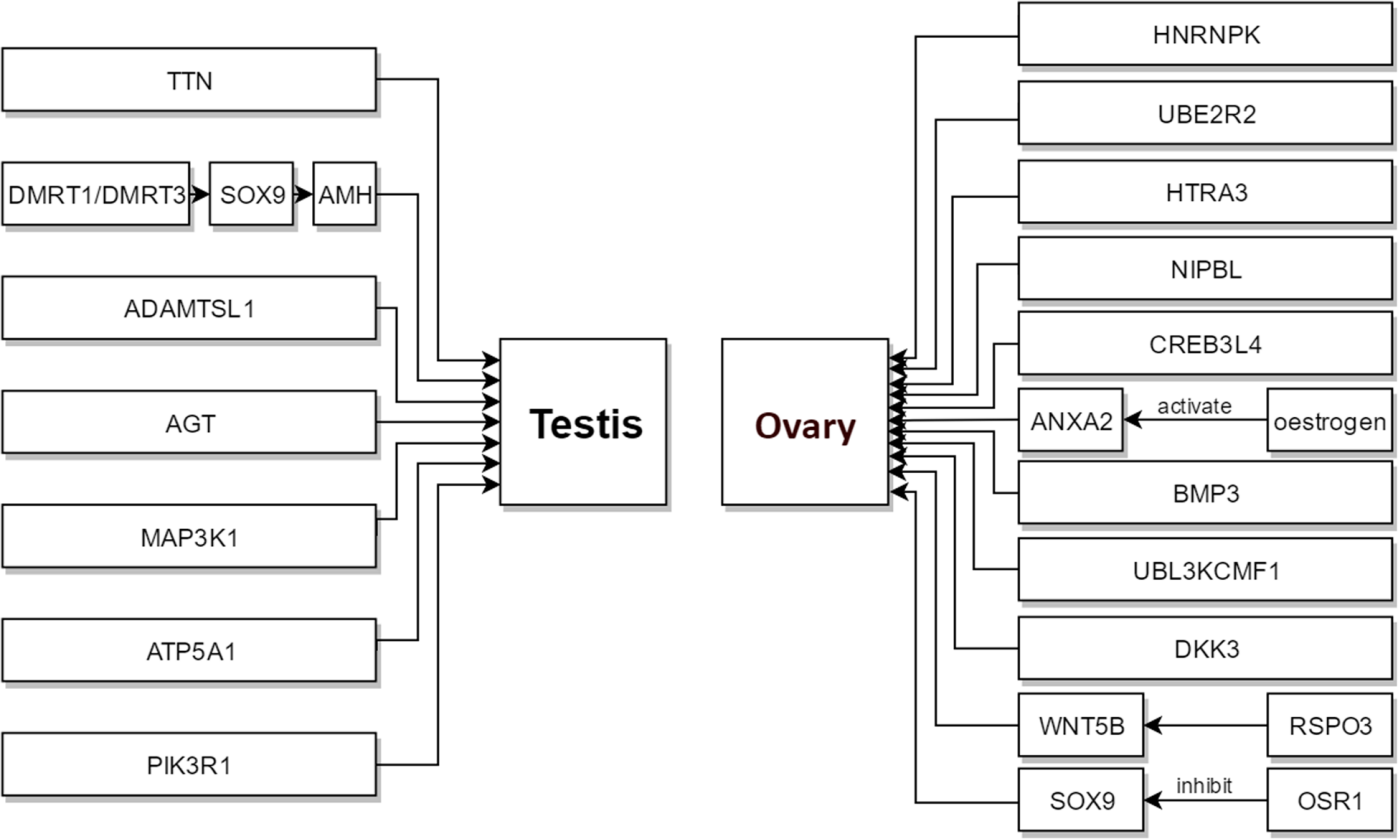
A speculation explaining the gene expression pattern in testes and ovaries

Little information has been reported regarding the reproductive activity and molecular aspects of gonadal development in Muscovy ducks. The transcriptome data generated herein provide valuable genetic resources to understand sex differentiation mechanisms and pathways underlying Muscovy duck reproductive regulation. A total of 315 DEGs were obtained, and a total of 146 signalling pathways were enriched from MB, FB, MA, and FA. DEGs were screened by clustering, GO analyses, and the published literature. We obtained 21 sex-related genes (Fig. 10).

Sex differentiation is suggested to be controlled by a major gene. *AMH* was found to be involved in gonad development and sex differentiation in this study. The *anti-Mullerian hormone type 2 receptor (AMHR2)* (the first AMH receptor identified in birds) is expressed in the early stage of sex differentiation and regulated by *DMRT1*, and *AMH* plays a decisive role downstream of *DMRT1* during sexual differentiation in birds [29]. In addition, *AMH* is significantly upregulated in MA vs FA and MA vs MB, that is, *AMH* is expressed in males after gonadal differentiation, so it is a candidate gene for the testis in Muscovy ducks. *DMRT1* was significantly upregulated in MA vs FA. Previous studies in chickens have identified *DMRT1* as a potential male-determining gene, which is expressed prior to *SRY-box transcription factor 9 (Sox9*) [30]. *DMRT1* is a requirement for male-specific expression and testicular development in vertebrates [31, 32]. Research in *Takifugu rubripes* [33] revealed that the expression levels of *DMRT1* and *DMRT3* is significantly higher in testis than in ovaries. Hence, *DMRT1* is associated with normal testicular development in Muscovy ducks. *DMRT3* was significantly upregulated in MA vs FA and MA vs MB. Previous studies in chickens have shown that *DMRT1* and *DMRT3* may bind to the enhancer of *SOX9* [30]. In addition, studies have found that *hemogen (HEMGN)* may activate *SOX9*. *HEMGN*, as a developmental window, is expressed after *DMRT1* and before *SOX9* [8]. We speculated that *DMRT1* and *DMRT3* induce gonads to develop as males in Muscovy ducks. Additionally, the expression of *DMRT1* and *DMRT3* induces *SOX9*, which then induces *AMH*.

In animals, spermatogenesis is a conserved process in which male diploid spermatogonia develop to maturity and haploid spermatozoa capable of fertilizing an oocyte [34]. Sperm individualization is one of the last stages of spermatogenesis, which separates spermatids from syncytial male germline cysts [35]. *TTN* was involved in male gamete generation, spermatogenesis, the sperm individualization process, and the germ cell development process in this study. *Titin (TTN)* plays key structural, developmental, mechanical, and regulatory roles in cardiac and skeletal muscles [36]. In *Bombyx mori*, *Bmkettin (TTN)* is expressed at higher levels in males than females [37, 38]. *TTN* was significantly upregulated in MA vs FA and MA vs MB. Thus, *TTN* was expressed in males after gonadal differentiation in Muscovy ducks.

Fertilization is a multi-step process that culminates in the fusion of the sperm and oocyte plasma membranes [39]. Herein, *AGT* was involved in fertilization and the acrosome reaction process. In the kidney cortex, AGT gene expression was higher in males than females [40, 41]. Moreover, both *SRY* and *SRY-box transcription factor 3 (SOX3)* upregulated the promoter of *AGT* [42]. In addition, *AGT* was significantly upregulated in MA vs FA. We speculated that *AGT* was associated with normal testicular development in Muscovy ducks.

During oogenesis, gene expression of oocytes is dynamically regulated by a series of well-coordinated transcription factors that are active in germ cell lines and somatic cells. Many somatic-expressed and germ cell-specific transcriptional regulators play key roles in folliculogenesis and ovarian formation [43]. Here, *BMP3* was involved in oogenesis, the female gamete generation and germ cell development process. Gametogenesis begins with an early germline distribution in the embryo [43]. During development, both male and female germ cells undergo a round of meiotic cell division to reduce the ploidy of the gametes, and thus maintain the ploidy after fertilization [44]. In the chicken embryo, *BMP3* is preferentially expressed in the developing ovary [45]. *BMP3* was significantly upregulated in FA vs FB. Therefore, in Muscovy ducks, *BMP3* induced differentiated gonads to develop as females.

The aromatase enzyme is a key enzyme in the regulation of oestrogen levels [46]. Oestrogens not only appear in the form of female hormones [47], but also as steroids that are necessary for the endocrine balance in males [48]. Our present results showed that *ATP5A1* and *OSR1* were involved in cellular aromatic compound metabolic processes, and *NIPBL*, *HNRNPK*, *ANXA2* and *CREB3L4* were involved in aromatic compound biosynthetic processes. *ATP5A1* exists in a copy on the non-recombinant region of the Z and W chromosomes [10, 49, 50]. *ATP5A1* is a Z-linked gene in chickens [51]. Thus, *ATP5A1* was expressed in male Muscovy ducks. *OSR1* and *ANXA2* were upregulated both in FA vs MA and FA vs FB. Thus, *OSR1* and *ANXA2* were expressed in males after gonadal differentiation. As a member of the OSR family, *OSR1* is a zinc finger transcription factor that is required for embryonic development [52]. OSR1 is enriched in the pregranular cell population of females, indicating that the supporting cell progenitors are female-primed before gonadal sex differentiation in the chicken embryo [14]. *OSR1* can bind to the promoter region of *SOX9*, and overexpression of *OSR1* can inhibit the expression of *SOX9* [53]. Therefore, we speculated that although *OSR1* was not directly involved in sex differentiation, it affected sex differentiation by inhibiting the expression of *SOX9* in ovaries. *ANXA2* plays a role in the development of chicken ovaries and follicles [54]. Relevant studies have reported that oestrogen may stimulate *ANXA2* expression [55]. In brief, *OSR1* and *ANXA2* were associated with the differentiation of ovaries in Muscovy ducks. *HNRNPK* interacts with DNA, RNA, and various proteins to participate in transcription, translation, RNA splicing, DNA repair, and chromatin remodelling, and it plays key roles in the nervous system. *HNRNPK*, as an oestrogen-induced transcription factor, is involved in ovary development [56–60]. *NIPBL*, as a sex-related gene in chickens, is assembled on the W chromosome that has homologous sequences on chromosome Z [61]. *CREB3L4* is characterized by a unique putative transmembrane domain with its C-terminus arranged in the bZIP region, which is assigned to the CREB/ATF subfamily transcription factors [62, 63]. In addition, in mice, *CREB3L4* is expressed in a stage-specific manner during sperm cell differentiation, and the lack of *CREB3L4* does not dramatically affect mouse spermatogenesis [64, 65]. *NIPBL, HNRNPK* and *CREB3L4* were significantly upregulated in FB vs MB and FA vs MA, and we analysed sex differentiation under this condition. We found that *NIPBL, HNRNPK* and *CREB3L4* were expressed in female Muscovy ducks.

In our study, *ADAMTSL1* and *PIK3R1* were significantly upregulated in MA vs FA and MA vs MB. Previous studies have indicated that *ADAMTSL1* lies on the Z chromosome and is highly expressed in the testis during sex differentiation in chicken embryos [45]. *PIK3R1*, an essential regulatory gene in the phosphatidylinositol 3-kinase/AKT (PI3K/AKT) signalling pathway, engages in various biological activities and metabolic pathways in organisms [66, 67]. In adult half-smooth tongue sole, the expression of *PIK3R1* in male gonads has been reported to be significantly higher than that in other tissues [68]. Therefore, *ADAMTSL1* and *PIK3R1* were expressed in males after gonadal differentiation and related to testicular development in Muscovy ducks. *MAP 3 K1* was significantly upregulated in MA vs FA. previous studies have shown that knocking out *MAP 3 K1* results in testicular abnormalities in developing mice [69]. Additionally, mutations in *MAP 3 K1* downregulate testicular decisive factors such as *SOX9* and *SRY* [70]. Therefore, *MAP 3 K1* is associated with normal testicular development in Muscovy ducks.

In this assay, *HTRA3* was upregulated both in FA vs MA and FA vs FB. *DKK3* was significantly upregulated in FA vs FB, and *RSPO3* was significantly upregulated in FA vs MA. *HTRA3* is an important reproduction-related gene and regulates ovarian development [71–74]. *HTRA3* inhibits TGF-β signalling during mouse embryonic development [75]. *RSPO3* is a member of the RSPO protein family. Previous studies have shown that four members of the RSPO protein family can activate the WNT/β-catenin signalling pathway [76]. Furthermore, studies on mammals have also suggested that the WNT signalling pathway plays an significant role in ovarian development and hormone secretion [77, 78]. Moreover, *RSPO3* is more effective in activating the WNT/β-catenin signalling pathway [79]. *Dkk3* is abundant in the liver and brain [80]. Moreover, as a soluble Wnt inhibitor, most knowledge about *Dkk3* involves molecular cancer therapy**.***Dkk3* may be an activator or inhibitor of the canonical pathway [81–89], which is strictly context-dependent [90]. In this study, *DKK3* can positively or negatively regulate the Wnt signalling pathway. Additionally, *DKK3* was enriched in biological process with the regulation of female receptivity. The functions and expression of *DKK3* were also associated with ovary development [91]. Therefore, *HTRA3, DKK3* and *RSPO3* were associated with the differentiation of ovaries in Muscovy ducks.

In addition to known sexual regulators based on previous studies in other species, *WNT5B, UBE2R2* and *UBL3KCMF1* were identified. *WNT5B* was significantly upregulated in FA vs MA and FA vs FB. Thus, *WNT5B* was expressed in females after gonadal differentiation. Moreover, *WNT5B* was enriched in the WNT/β-catenin signalling pathway, and research has shown that *RSPO3* is more effective in activating the WNT/β-catenin signalling pathway [79]. As a result, we hypothesized that *RSPO3* is involved in the differentiation of ovaries in Muscovy ducks by activating *WNT5B* in the WNT signalling pathway. *UBE2R2* and *UBL3KCMF1* were significantly upregulated in both FB vs MB and FA vs MA. Furthermore, these genes were associated with the ubiquitin-binding enzyme E2 and ubiquitin protein ligase E3, respectively. It has been reported that the expression level of ubiquitin-binding enzyme E2 is significantly different at different developmental stages of the testes and ovaries [92]. Previous studies have demonstrated that ubiquitin conjugating enzyme 9 (Ubc9), a member of the ubiquitin-binding protein E2 family, is implicated in the regulation of oogenesis [93]. These two genes showed higher expression in female than male gonads before and after sex differentiation. *UBE2R2* was assigned to the W and Z chromosome in chicken [94, 95]. As a result, *UBE2R2, UBL3KCMF1* and *WNT5B* were associated with the differentiation of ovaries in Muscovy ducks.

## Conclusions

In this study, RNA-Seq revealed 101.76 Gb of clean reads and 2800 DEGs, including 46 in MB vs FB, 609 in MA vs FA, 1027 in FA vs FB, and 1118 in MA vs MB. A total of 146 signalling pathways were enriched by KEGG analysis. Furthermore, a total of 21 candidate genes related to sex differentiation and development in Muscovy ducks were screened by clustering and GO analyses. Among these, 12 genes, namely, *UBE2R2, UBL3KCMF1 RSPO3, WNT5B, BMP3, HNRNPK, NIPBL, CREB3L4, HTRA3, DKK3, ANXA2* and *OSR1*, were involved in the differentiation and development of ovaries. Moreover, 9 genes, namely, *DMRT1, DMRT3, AMH, MAP3K1, PIK3R1, TTN, ATP5A1, AGT* and *ADAMTSL1*, were related to the differentiation and development of testes. Furthermore, after gonadal differentiation, *DMRT3, AMH, PIK3R1, ADAMTSL1, AGT* and *TTN* were especially highly expressed in males, and *WNT5B, ANXA2* and *OSR1* were especially highly expressed in female. These results provide a reference for subsequent research on the mechanism of sex differentiation in Muscovy ducks and significant information for the study of sex control in Muscovy ducks.

## Methods

### Ethics statement

This study adhered to the recommendations of the “Regulations for the Management of Affairs Concerning Experimental Animals” (Ministry of Science and Technology, China, revised in June 2004). The Muscovy duck shed and feeding conditions were reviewed and approved by the Institutional Animal Care and Use Committee of the College of Animal Science, Fujian Agriculture and Forestry University. In this assay, all ducks were permitted continuous access to standard commercial feed rations and water. All efforts were made to minimize animal suffering.

### Animals and tissue collection

A total of 96 Muscovy duck eggs were provided by China Putian Guangdong Wenshi Poultry Co., Ltd. (Putian, China). Four groups were assessed (*n* = 3 per group): males and females at 8 days and 12 days of incubation, respectively. Eight paired gonads were pooled for each replicate. Muscovy eggs in good condition were examined at 8 days and 12 days of incubation. The selected Muscovy duck eggs were all from the same population and hatched at approximately 37 °C with a humidity of 60–70%. First, the selected duck egg was gently tapped with tweezers at the air chamber side, and the eggshell and shell membranes were removed to expose the embryo. The embryo was removed with a spoon, and then the embryonic gonad of the duck (including the kidney and gonads) was excised, labelled, and placed in liquid nitrogen. After all the samples were collected, they were stored at − 80 °C. The embryo head and tail were collected, and DNA was extracted for sex determination by PCR [12].

### RNA extraction and sequencing

RNA extraction was performed using TRIzol reagent in accordance with the manufacturer’s protocol. Then, 1% agarose gels were used to evaluate RNA degradation and contamination. RNA concentration and integrity were checked with an RNA Assay Kit and a Qubit® 2.0 Fluorometer and the RNA Nano 6000 Assay Kit for the Bioanalyzer 2100 system, respectively. Gonad tissue was pooled based on sex and stage of sex differentiation, eight gonads of the same genetic sex were mixed in a library, and a total of 12 libraries from the MB, FB, MA, and FA groups were sequenced. First, twelve libraries were constructed. Briefly, purification of mRNA and cleave the mRNA into fragments were performed. Then reverse transcription, cDNA synthesis, and cluster generation were carried out. The libraries were then sequenced on an Illumina HiSeq platform. These data are available at the NCBI Sequence Read Archive (accession number PRJNA625194, SRX8108890, SRX8108889, SRX8108888, SRX8108887, SRX8108886, SRX8108885, SRX8108884, SRX8108883, SRX8108882, SRX8108881, SRX8108880 and SRX8108879).

### Bioinformatic analysis

First, after removing reads containing adaptors, poly-N sequences and low-quality reads from the raw data through in-house Perl scripts., clean reads were obtained. Next, all the high-quality clean reads were aligned to the *Anas platyrhynchos* genome using Hisat2 v2.0.4. Subsequently, HTSeq v0.9.1 and the fragments per kilobase of transcript per million mapped reads (FPKM) were used to quantify gene expression level [96, 97]. In addition, differential expression analysis of MB vs FB, FA vs FB, MA vs FA, and MA vs MB was performed using the DEGSeq R package (1.18.0). We used the Benjamini & Hochberg method corrected *P*-value < 0.05 as the threshold to consider to be the differentially expressed genes. The GO and KEGG enrichment analyses were then conducted according to a P-value cut-off of 0.05 using GOSeq Release2.12 and KOBAS v2.0 [98, 99]. Finally, Hisat2 v2.0.4 was used as the mapping tool, and reads without annotation information were assembled with the Cufflinks v2.1.1 reference annotation-based transcript (RABT). Cuffcompare was compared with known genetic models to obtain new genes [97, 100].

### Real-time PCR verification

Nine significantly DEGs were selected for real-time quantitative PCR (qRT-PCR) to validate the RNA-Seq results. The PrimeScript™ RT reagent Kit with gDNA Eraser was used to cDNA synthesis following the manufacturer’s instructions. The primer information for these genes and the endogenous reference gene are available in Table 3. β-actin was employed as the endogenous reference gene. The qPCR mixture consisted of 10 μL of 2 × Master Mix, 2 μL of cDNA, 0.5 μL of each primer and 7 μL of ddH_2_O under the following conditions to collect fluorescence: denaturation at 95 °C for 30 min, followed by 40 cycles of amplification at 95 °C for 5 s and 60 °C for 40 s. To establish the melting curve of the PCR product, the reaction was performed at 95 °C for 10 s, 60 °C for 60 s, and 95 °C for 15 s, after which it was slowly heated from 60 °C to 99 °C. The relative expression levels of target genes in each sample were individually determined using the 2-ΔΔCT method [101].

## Supplementary information

**Additional file 1.**

## Data Availability

The datasets generated and analysed during the current study are available in the NCBI Sequence Read Archive database (https://www.ncbi.nlm.nih.gov/sra), under the accession number PRJNA625194 (https://www.ncbi.nlm.nih.gov/bioproject/PRJNA625194), SRX8108890, SRX8108889, SRX8108888, SRX8108887, SRX8108886, SRX8108885, SRX8108884, SRX8108883, SRX8108882, SRX8108881, SRX8108880 and SRX8108879. The name of the bioproject is Muscovy ducks sequencing. The link to the *Anas platyrhynchos* genome is https://www.ncbi.nlm.nih.gov/genome/?term=Anas+platyrhynchos. The primer information for β-actin (GeneBank accession number EF667345.1) is available in Table 3.

## Abbreviations

MB: Male before gonadal differentiation
FB: Female before gonadal differentiation
MA: Male after gonadal differentiation
FA: Female after gonadal differentiation
DEGs: Differentially expressed genes
DMRT1: Doublesex and mab-3 related transcription
SRY: Sex-determining Region on the Y Chromosome
CYP19A1: The cytochrome P450 family 19 subfamily A member
AMH: Anti-Mullerian hormone
E: Embryonic day
ESR-α: Oestrogen receptor-α
ATP5A1: ATP synthase, H+ transporting, mitochondrial F1 complex, alpha subunit 1
TTN: Titin
NIPBL: Nipped-B-like protein
CREB3L4: Cyclic AMP-responsive element-binding protein 3-like protein 4
HNRNPK: Heterogeneous nuclear ribonucleoprotein K
HEMGN: Hemogen
SOX9: SRY-box transcription factor 9
SOX3: SRY-box transcription factor 3
Go: Gene Ontology
KEGG: Kyoto Encyclopedia of Genes and Genomes
BP: Biological process
CC: Cellular component
MF: Molecular function
MAPK: The mitogen-activated protein kinases
TGF-β: Transforming growth factor-β
DMRT3: Doublesex and mab-3 related transcription factor 3
HTRA: high-temperature requirement factor A
HTRA3: HtrA serine peptidase 3
OSR1: Odd-skipped related transcription factor 1
WNT5B: Wnt family member 5B
NKX2-1: NK2 homeobox 1
AMHR2: Anti-Mullerian hormone type 2 receptor
MAP3K1: Mitogen-activated protein kinase kinase kinase 1
PI3K: Phosphatidylinositol 3-kinase
PIK3R1: phosphoinositide-3-kinase regulatory subunit 1
ADAMTSL1: A disintegrin and metalloproteinase with thrombospondin motif like 1
UBE2R2: Ubiquitin-conjugating enzyme E2 R2
UBL3KCMF1: E3 ubiquitin-protein ligase KCMF1
Ubc9: Ubiquitin conjugating enzyme 9
RSPO3: R-spondin 3
ANXA2: Annexin A2
RIN: RNA integrity number
FPKM: Fragments per kilobase of transcript per million mapped reads
RABT: Reference Annotation Based Transcript
qRT-PCR: real-time quantitative PCR
